# Immunomodulation by Gut Microbiome on Gastrointestinal Cancers: Focusing on Colorectal Cancer

**DOI:** 10.3390/cancers14092140

**Published:** 2022-04-25

**Authors:** Raghad Khalid AL-Ishaq, Lenka Koklesova, Peter Kubatka, Dietrich Büsselberg

**Affiliations:** 1 Weill Cornell Medicine-Qatar, Education City, Qatar Foundation, Doha 24144, Qatar; rkmalishaq@hotmail.com; 2 Department of Obstetrics and Gynecology, Jessenius Faculty of Medicine, Comenius University in Bratislava, 036 01 Martin, Slovakia; koklesova5@uniba.sk; 3 Department of Medical Biology, Jessenius Faculty of Medicine, Comenius University in Bratislava, 036 01 Martin, Slovakia; peter.kubatka@uniba.sk

**Keywords:** gut microbiome, immune system, immune–gut interaction, gastrointestinal cancer, colorectal cancer, anti-cancer

## Abstract

**Simple Summary:**

A symbiotic relationship with the host gut microbiome influences the immune system’s development, functions, and activities. In the mucosa, the gut microbiome mediates several immune activities such as the induction of naïve T-cells differentiation, production of cytokines, and myeloid cells activation. The gut-immune interaction and GI cancer development were investigated more recently. Understanding the interaction’s underlying mechanism provides insight to use them as potential anti-cancer targets. Even though multiple reports support the role of gut-immune interactions in targeting cancer-related pathways such as inflammation, apoptosis, and cellular proliferation, efforts are required to assess their interaction and impact on current treatment options.

**Abstract:**

Gastrointestinal cancer (GI) is a global health disease with a huge burden on a patient’s physical and psychological aspects of life and on health care providers. It is associated with multiple disease related challenges which can alter the patient’s quality of life and well-being. GI cancer development is influenced by multiple factors such as diet, infection, environment, and genetics. Although activating immune pathways and components during cancer is critical for the host’s survival, cancerous cells can target those pathways to escape and survive. As the gut microbiome influences the development and function of the immune system, research is conducted to investigate the gut microbiome–immune interactions, the underlying mechanisms, and how they reduce the risk of GI cancer. This review addresses and summarizes the current knowledge on the major immune cells and gut microbiome interactions. Additionally, it highlights the underlying mechanisms of immune dysregulation caused by gut microbiota on four major cancerous pathways, inflammation, cellular proliferation, apoptosis, and metastasis. Overall, gut-immune interactions might be a key to understanding GI cancer development, but further research is needed for more detailed clarification.

## 1. Introduction 

### 1.1. Gastrointestinal Cancer 

Globally, cancers are a significant cause of death and disability [1]. They are characterized by impaired homeostasis and cellular functions [2]. Cancers are classified based on the organ, tissue of origin, or the cancer cell’s molecular characteristics [3] and the development of cancers is influenced by environmental and genetic factors such as obesity, diet, smoking, and infections with pathogenic agents [4]. Gastrointestinal cancers (GI) are considered a major public health problem with challenging economic and medical burdens due to their high prevalence and mortality rate [5]. The symptoms and signs of GI cancers depend on the type of cancer (gastric cancer (GC), colorectal cancer (CRC), esophageal cancer (EC), pancreatic cancer (PC), and hepatocellular carcinoma (HCC)). They might include weight loss, abdominal pain, dysphagia, and anorexia [6] and the progression of GI cancers occurs in a multistage process. They result from uncontrolled cellular proliferation, the loss of apoptotic functions through the intrinsic and extrinsic apoptotic pathways, and the impairment of major pathways such as epithelial–mesenchymal transition (EMT), phosphatidylinositol 3-kinase (PI3K)/protein kinase B (AKT), and nuclear factor-kappa (NF-κB) signaling pathways [7,8]. Efforts are required to understand GI-cancers’ underlying mechanisms through these specific impaired pathways.

### 1.2. The Immune System in Cancer Pathogenesis 

The human immune system is defined as a group of cells that protect the body from foreign antigens such as toxins, microbes, viruses, and cancer cells [9]. The immune system has two lines of defense that complement each other; innate and adaptive immunity. Imbalance or defects in either line of defense could result in an inappropriate immune response in the body [10]. 

Cancer and the immune system have been widely discussed for a century [11]. The underlying mechanism between cancer cells and the immune system interaction involves three processes of how the immune system defends and protects the host; (i) the identification of non-self cells, (ii) the production of effector cells to specifically target the cancerous cells, and (iii) the development of immunological memory as a defense mechanism [12]. The role of immune cells in cancer includes both a pro-tumorigenic and an anti-tumorigenic function [11]. Inflammatory immune cells activation in cancer can present in different tumorigenesis stages and can lead to epigenetic modification, the induction of cancerous cellular proliferation, genomic instability, and the enhancement of a cancerous anti-apoptotic pathway, therefore, leading to cancer progression and dissemination [11]. During the pathogenesis of cancer, multiple components and pathways of innate and adaptive immunity are activated to identify cancerous cells and target their genetic and epigenetic alterations and modifications, thus leading to cancer elimination [13]. Such pathways include complement proteins activation aiding in cancer eradication, natural killer (NK) cells, cytotoxic immune cells which recognize and eliminate immunogenic cancerous cells, neutrophil protease activation, anti-tumor macrophages which display a pro-inflammatory like polarization playing a role in the elimination of immunogenic cancerous cells, CD4+ T-cells activation, the production of IL-22 promoting T-cells proliferation, and naïve B cells activation [14,15]. Despite these mechanisms, cancer can manage to overcome immune components as in the case of T-cells, in which cancerous cells can impair the functions of anti-tumor T-cells such as their ability to infiltrate the tumor survival, cytotoxicity, and proliferation abilities [15]. 

Advances in the development of immuno-oncology have changed the treatment of GI cancer. Multiple ongoing clinical trials evaluate the efficacy and safety of immunotherapy agents such as avelumab (anti-PD-L1) and relatlimab (anti-LAG3) in patients with advanced gastric cancer [16]. Additionally, as for CRC, two immune checkpoint inhibitors target programmed death-ligand 1 (PD-1) in metastatic cancer, namely, KEYNOTE 028 and CheckMate 142, with an objective response rate of 40% and 55%, respectively [17]. More studies are required to identify the common side effects of these treatments, to estimate the impact on patients with immunodeficiency, and to evaluate the role of gut microbiota in treatment utilization. 

### 1.3. Gut Microbiota: Role in GI Cancer Immunity 

In the human body, trillions of microorganisms, such as bacteria, viruses, fungi, and protozoan, are known as the microbiota [18]. The microbiota resides mainly on the respiratory and gastrointestinal tract’s mucosal surfaces with different concentrations and relative abundances [19]. Over time, changes in the microbiome composition occur due to internal or external factors such as lifestyle, genetics, geographical locations, and age, leading to significant variations between individuals [20]. The gut microbiome plays a role in protection from infections, vitamin production, and immune cells development and activity [21], but intestinal dysbiosis and the imbalance in the number of microbes and their diversity in the gut is linked to several pathogeneses such as cancer [22]. 

Studies have reported the impact of microbiota on the development, activities, and function of immune cells [23]. In mucosal sites, where the microbiota prominently resides, early B-lineage cells development occurs under the influence of extracellular signals from the microbiota [24]. Additionally, the gut microbiome promotes the differentiation of naïve T-cells into colonic Treg cells with unique T-cell receptors on their surfaces [25]. During the invasion of pathogenic bacteria, the gut microbiome promotes the activation of myeloid cells leading to cytokines production [23]. 

**Figure 1:**
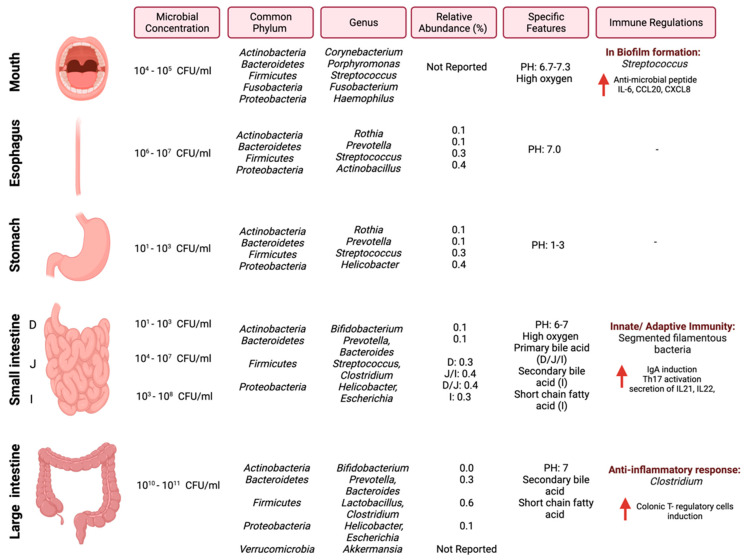
Schematic illustration of regional diversity of the microbiome along the GI tract. The figure is divided into different regions of the GI tract and highlights the microbial concentration ranges, common phylum and genus, relative abundance [27], and immune regulations specific to each region. “Created with BioRender.com”.

In cancer, the gut microbiome influences the anti-tumor immune response through (1) the induction of the T-cells response, (2) the engagement of a pattern recognition receptor that has pro-inflammatory effects, or (3) the mediation of specific metabolites, which can activate T-cell receptors [26]. Efforts are required to investigate and understand the underlying mechanisms between the gut microbiome and the immune system in the context of cancer and how those mechanisms can be utilized as targets for cancer therapy. Figure 1 summarizes the most common pathogens in the GI tract, their relative abundance, and reported immune regulations. 

This review has analyzed published studies that report the crosstalk between the gut microbiota and the immune system, assessing the impact of this communication on specific GI cancer pathways. Additionally, it identifies gaps in the current literature. 

## 2. Search Strategy and Selection Criteria 

Using the databases “Medline”, “Scopus”, and “PubMed”, papers published from 2001 were searched, using the search terms “Immune cells”, “microbiota”, “Immune cells AND microbiota”, “microbial metabolism”, “Innate immunity AND microbiome”, “Microbiome AND GI cancer”, “gut microbiota enzymes”, “gut microbiome AND immune cells AND GI cancer”, “gut microbiome AND immune cells AND gastric cancer”, and “gut microbiome AND immune cells AND colorectal cancer”. The search yielded around 2000 articles, and in this article, we selected 166 articles and analyzed them in detail. Duplicate studies were excluded, and eligible studies were selected based on inclusion and exclusion criteria. The inclusion criteria included papers that discussed gastrointestinal cancer models or tissues and highlighted gut microbiome interactions. 

## 3. Microbiota–Immune Interactions

**Figure 2:**
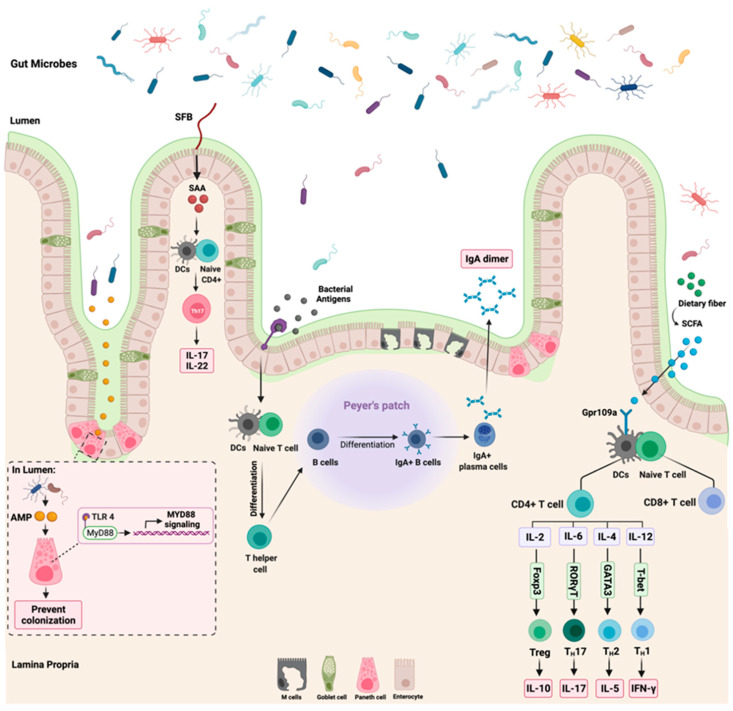
Summary of the most reported gut microbiome and immune interactions. While the left side of the figure illustrates the influence of segmented filamentous bacteria (SFB) and antimicrobial peptides (AMP) on the host immune system through the activation of Th17 and MyD88 signaling, respectively, the right section of the figure highlights the role of dietary fibers and short chain fatty acids (SCFA) on T cells expression. The role of bacterial antigens on the production of IgA dimer is shown in the middle part of the figure. “Created with BioRender.com”.

The colonization of the gut with microorganisms led to physiological adaptation in the body, as seen with immune cells development, maturation, and interaction [28]. The relationship between the microbiota and the human body is tightly regulated through a controlled immune response to avoid immune activation that might harm the body [29]. This section will discuss three major interactions between the gut microbiome and the immune system: (1) segmented filamentous bacteria (SFB), (2) antimicrobial peptides, and (3) dietary fibers such as short-chain fatty acids (SCFA). Figure 2 highlights these three interactions.

### 3.1. Segmented Filamentous Bacteria 

SFB are commensal bacteria found mainly in the small intestine [30]. They are gram-positive bacteria identified by their long and filamentous appearance [31]. A genome sequencing listed SFB as a member of *Clostridiales*. Additionally, the sequencing results reported a lack of amino acid biosynthetic enzymes in SFB and an expression of typical flagella and spore-forming genes. This suggests that those bacteria depend on the host for essential nutrients [32]. 

The colonization of SFB in the intestine regulates and influences the immune response in the body [33]. SFB regulates the level of IL-17A and IL-22 expression in the intestine through the modulation of serum amyloid A (SAA) [34]. Additionally, SFB play a role in postnatal maturation of the immune system through the production of IL-17 producing CD4+ T-cells, which is critical in host protection against extracellular pathogens [35]. The observed effects of SFB on the immune system occur due to their ability to adhere to the intestinal epithelium, which is a crucial step to induce Th17 cells differentiation. SFB models lacking the ability to adhere failed to induce intestinal Th17 differentiation [36]. Following the adherence of SFB to the intestinal epithelium is the secretion of SAA, which is essential for cytokines production and secretion [37]. Despite what is known so far about the role of SFB in shaping the intestinal immune response, more research is still required to understand the interaction of SFB with other microbiomes such as viruses and how they all impact the immune system. Additionally, the mechanism in which SFB modulates the immune system requires further understanding as well as how distal organs react to those immune changes modulated by SFB.

### 3.2. Short Chain Fatty Acids 

SCFAs are fermented fatty acids generated by the gut microbiota, such as *Faecalibacterium prausnitzii*, from the digestion of complex carbohydrates [38]. They are considered the most abundant microbial-derived metabolites in the human gut lumen. They consist mainly of propionate, butyrate, and acetate [39]. SCFAs play a critical role in improving the function of the gut barrier, protecting against microbial invasions, and reducing intestinal inflammation, thus improving the host’s overall health status [40]. Those observed positive effects of SCFAs are due to the activation of G-protein coupled receptors (GPCRs) such as GPR109a or the suppression of histone/histone deacetylases (HDACs) which influence genetic expression [41]. 

In colonocytes, the sodium-dependent monocarboxylate transporter-1 (SLC5A8) facilitates and mediates the entry of SCFAs (specifically butyrate) from the lumen to the colonic epithelial cells. This leads to the suppression and activation of HDACs and GPCRs, respectively [42]. SCFAs are essential regulators of immune cells’ recruitment, activation, and differentiation, such as dendritic cells (DC), neutrophils, macrophages, and T-lymphocytes. Additionally, SCFAs regulate the expression of pro-inflammatory cytokines such as IL-12 and IL-6 [43]. Moreover, the binding of butyrate to GPR109a receptor on DCs results in an increased expression of IL-10 and a decreased expression of IL-6, which results in increased T-reg cells development, thus inhibiting Th17 cells expansion [44]. This indicates that the GPR109a receptor is vital in anti-inflammatory pathways such as apoptosis, especially in inflammation-induced colon cancer [45]. Figure 2 summarizes the reported interaction between butyrate and GPR109a and the subsequent cytokines production. Despite the observed tumor suppressor effects of GPR109a receptors, some reports highlighted that the activation of this receptor leads to the activation of inflammatory signaling pathways, which suggest that GPR109a could act as a tumor activator and suppressor depending on the affected sites and tissues [46]. Further research is required to investigate the effect of the GPR109a receptor and other GPCRs receptors. Additionally, more efforts are necessary to understand the effect of other SCFAs such as acetate on the host immune system. 

### 3.3. Antimicrobial Peptides 

The intestine contains many microorganisms that provide multiple benefits in metabolism, nutrients, and immunity [47]. The symbiotic relationship between the host and the gut microbiota is mediated by chemical and physical gut mucosal barriers, preventing unregulated interaction between the host immune system and the gut microbiota [48]. Antimicrobial peptides (AMPs) are considered a chemical mucosal barrier of basic amino acid-rich proteins with a broad spectrum of antimicrobial properties, such as being cytolytic, microbicidal, and bacteriostatic [49]. Shortly after infection, AMPs are synthesized promptly to rapidly neutralize the invading microbes [50]. Additionally, AMPs include the defensin protein family, such as alpha and beta. They bind to the microbial cell membrane and disrupt the membrane integrity by forming pore-like structures. Deficiency in alpha defensin, a highly expressed protein in Paneth cells, is associated with gut microbiota alteration suggesting that AMPs play a role in gut environment hemostasis [51]. Moreover, patients with inflammatory bowel disease have reported having intestinal barrier dysfunction as the level of AMPs production was reduced [52]. 

The Toll-like receptor (TLR) family plays a role in enhancing the function of the epithelial barrier and innate immunity [53]. After an infection, TLRs recognize the synthesized bacterial products such as AMPs which activate the cytoplasmic adaptor protein MyD88 [54]. Activated MyD88 is essential in protecting the intestinal epithelial cells from mucus-associated bacteria and opportunistic bacteria. Additionally, a loss of MyD88 signaling activation can disrupt microbiota and host tissue segregation, compromise epithelial barrier function, and alter the balance of the gut microbiota community [55]. Moreover, TLRs are critical for IgA antibody secretion. After producing bacterial antigens, the TLRs sense those antigens, leading to T-cell differentiation and subsequently producing IgA dimers. The production of IgA is essential to distinguish between pathogenic and commensal bacteria where it neutralizes the mobility and adhesions of the pathogenic bacteria [56]. Figure 2 summarizes the role of TLRs in MyD88 signaling activation and IgA production.

## 4. Microbiota–Immune Interactions: Role in GI Cancer Development

The gut microbiome plays a critical role in the pathogenesis of host diseases such as cancer [57]. As the gut microbiome is influenced by several factors such as diet, genetics, and lifestyle, its dysbiosis, either in the bacterial composition, bacterial bioactivity or diversity, can impair the balance of specific bacterial species and increase the abundance of inflammation-inducing species that can cause several diseases including inflammatory bowel disease and cancer [58]. The gut microbiome influences the host immune response to regulate cancer mechanisms such as progression, genetic instability, and the response to treatment [59]. Animal studies have reported that specific microbes such as *Bacteroides fragilis* and *Escherichia coli* can promote cancer development by releasing genotoxins, damaging the host DNA [60]. Additionally, the gut microbiome could impact the efficacy of cancer treatment, as seen in antibiotic-treated mice [61]. This suggests the critical role of intact microbiota in the gut for optimal treatment outcomes. 

Additionally, the gut microbiome impacts the function of the mucosal B and T cells, which are essential for immune homeostasis as they inhibit the unregulated response to harmless antigens and preserve the mucosal barrier integrity in the intestine [62]. Disruption of the gut barrier facilitates the interaction between the immune cells and the microorganisms, resulting in cancer development through the induction of immunosuppressive or pro-inflammatory pathways [63]. Gut microbiome dysbiosis can influence cancer pathways by recruiting lymphocytes to the intestine, leading to cellular proliferation by activating the IL-6 pathway [64]. Moreover, TLRs upregulation can activate the nuclear factor (NF)-κB and JAK/STAT3, which are critical for immunosuppression and cellular proliferation [65]. Table 1 summarizes the main findings from the reported studies.

**Table 1:** Representative microbial–immune interactions and their underlying anticancer effects.

Targeted Cancer Pathway	Type of Cancer (s)	Microbial Species	Targeted Metabolites/ Proteins/ Genes/ Species	Targeted Immune Cells/ Pathways/ Products	Site of Interaction	Mechanism of Action	Methods of Testing	Model Used		References
								In Vivo	In Vitro	
Inflammation	Colon cancer	*Enterococcus* *Bacteroidetes* *Lactobacillus* *E. coli* Segmented filamentous bacteria	Short-chain fatty acids (SCFA)	IL-18 IL-6 IL-22	Colon Intestine	- SCFA receptors (GPR43, GPR41) promote barrier immunity - Suppress bacterial invasion - Regulate T-cell response in the intestine (TH17) - Promote the expression of intestinal tight junction proteins	Quantitative reverse transcription PCR Flow cytometry FISH Confocal microscopy	- C57BL/6 mice - Apc Min/+ mice		[66]
	Colon tumorigenesis	*Erysipelotrichaceae* *Prevotellaceae* *Lachnospiraceae*	Not specified	CD8 T cells IFN-γ IL-1β	Colon	- Gut dysbiosis promote tumorigenesis via CD8-independent mechanisms - Presence of specific bacterial populations - Gut dysbiosis promotes T cell exhaustion which reduces anti-tumor immunity	16S rRNA sequencing linear discriminant analysis (LDA) Quantitative reverse transcription PCR Antibiotic and antifungal studies Flow cytometry	- SPF WT1 mice - Cd8 mice		[67]
	Colon cancer	Mix of enteric flora from fecal samples	Compound K	IL-8	Colon	- Compound K exerts an anti-proliferative effect on colon cancer - Compound K actively inhibited the cellular growth of colon cancer - Compound K significantly induced apoptosis - Compound K significantly reduced the production of IL-8 at 20 μM - Compound K exerts significant anti-inflammatory effects on colon cancer at low concentration	Flow cytometry liquid chromatography quadrupole time-of-flight mass spectrometry ELISA		- HCT-116 - HT-19	[68]
	Colon cancer	Segmented filamentous bacteria *Proteobacteria* *Firmicutes*	FAM3D (cytokine like family) a gut secreted protein	CD3 T cells B220+ B cells CD11b+ myeloid cells	Colon	- FAM3D deficiency impaired mucosal barrier function by reducing acidic mucins expression. This leads to the expansion of potential pathogens - such as Deferribacteraceae and Muribaculaceae. - Absence of this molecule lead to increased low-level inflammation	Immunofluorescent staining Real-time PCR Western blot Quantitative reverse transcription PCR FISH	- C57BL/6 mice		[69]
	Colon cancer	*Bacteroidetes* *Prevotellaceae* *Firmicutes*	Gpr109a	IL-17 IL-23 ILC3	Colon	- Gpr109a suppresses IL-23 production by dendritic cells - IL-23 plays a role in the induction of inflammatory bowel disease - Gpr109a inhibits the production of microbiota-induced inflammatory cytokines	Antibody treatment Quantitative PCR Microbiome sequencing	- C57BL/6 mice		[70]
	Colon cancer	*Helicobacter hepaticus Lachnospiraceae*	TGF-β	NF-β	Colon	- Disruptions in the TGF-β signaling can cause tumorigenesis if combined with Helicobacter hepaticus - Deficiency in TGF-β leads to a decrease in butyrate production which can promote tumor formation and inflammation	DNA/RNA sequencing Multi-omics studies	- Smad3 mice		[71]
	Colon cancer	*Bacteroides* *Firmicutes*	IL-23 produced from dendritic cells	IL-1A IL-13 IL-17A CXCL-9 IL-17	Colon	- IL-23 level increased in colon cancer, and it correlates strongly with pro-inflammatory cytokines - IL-23 has a direct impact on epithelial barrier permeability - IL-23 is highly expressed in colon tumor samples - IL-23 triggers an inflammatory pathway through the Th17 expansion	Cell proliferation assays Cell migration and invasion assays ELISA Real-time PCR Ex-vivo studies Immunoblots	- F344 rats	- Caco2 - HCT116	[72]
	Colon cancer	*Prevotellaceae* Segmented filamentous Bacteria	LRP5/6-β-catenin-IL-10 signaling axis	TNF-α IL-6 IL-1β	Colon Intestine	- LRP5/6 signaling plays a role in suppressing colitis-associated tumor - Deficiency of LRP5/6 resulted in a marked increase in p38 MAPK activation, which is critical for the expression of inflammatory factors - LRP5/6 deficient mice displayed a higher level of CD4+ cells producing IL-17A compared to wild type mice - Deletion of LRP5/6 in CD11c+ APCs resulted in lower levels of IL-22 production	Antibiotic treatment Fecal microbiota transplant ELISA Cell sorting Flow cytometry Real-time PCR	- C57BL/6 mice - CD11c-cre mice		[73]
	Colon cancer	Not specified	TLR-4	Dual oxidase 2 (DUOX2) NADPH oxidase 1 (NOX1)	Colon	- The level of TLR4, DUOX2, and NOX1 was upregulated in colon cancer cells - Gut microbiota activate TLR-4, which stimulates ROS production through Duox2 even after the inflammation is treated - Activation of TLR4 and DUOX2 increases the production of H2O2, which promotes tumor initiation	Cell viability assays 16s ribosomal RNA polymerase chain reaction 16s ribosomal RNA sequencing	- Villin-TLR4 mice - C57Bl/6 mice		[74]
	Colon cancer	*Prevotella* *Escherichia coli* *Akkermansia* *Pseudoflavonifractor* *Ruminococcus* *Clostridium XlVa*	Short chain fatty acids (SCFA)	NOD-like receptor family pyrin domain containing 3 (NLRP3) Tumour necrosis factor-α (TNF-α) Interleukin-1β (IL-1β)	Colon	- Intestinal secretory immunoglobulin A (sIgA) expression was decreased in the mice receiving fecal samples from colorectal cancer patients - Real-time PCR results showed an upregulation in the expression of pro-inflammatory cytokines such as NLRP3, TNF-α, and IL-1β. - Gut microbiota from colorectal cancer patients enhanced the activation of Wnt signaling pathway	Fecal microbiota transplant Histological studies Immunohistochemistry staining Real-time PCR RNA extraction Western blotting RNA sequencing	- C57BL/6J mice	- Fecal samples	[75]
Cellular Proliferation	Colon cancer	*Fusobacterium nucleatum*	microRNA-31	CD3 T cells CD8 T cells CD45RO T cells FOXP3 T cells	Colon	- F. nucleatum arrested human T-cells in the G1 phase of the cell cycle - F. nucleatum expanded myeloid-derived immune cells, which can inhibit T-cells proliferation - MicroRNA-31 (miR-31) expression was significantly upregulated in cancer which can be associated with a poor prognosis	Quantitative PCR Ariol image analysis system Microarray Metagenomic analyses		- Colorectal carcinoma tissues from patients	[76]
	Colon cancer	*Bifidobacterium* *Prevotellaceae* *Bacteroides* *Lachnospiraceae*	YYFZBJS (traditional Chinese herbs)	CD4 T cells Foxp3 T-bet ROR-γt	Colon	- YYFZBJS reduced tumor multiplicity and numbers in the CRC mouse model - YYFZBJS treatment changed the composition of bacterial taxa in the colon - YYFZBJS induced multiple inflammatory pathways such as Treg/Th17 signaling leading to a significant expression of IL-6, IL-10, IL-17 - YYFZBJS inhibited cellular proliferation through Enterotoxigenic Bacteroides fragilis primed T-regulatory cells	Quantitative PCR Histology Genotyping Antibiotic treatment Fecal microbiota transplantation Flow cytometry Bacterial attachment assay	- ApcMin/+ mice	- HCT116 cells - MC-38 cells	[77]
	Colitis-associated colon cancer (CAC)	Not specified	TLR-4	TNF-a IL-1b	Colon carcinoma	- During the inflammatory phase of colon cancer, TLR-4 was upregulated in colonic tissues, which promoted tumor development - Blocking TLR-4 with TAK-242 reduced the release of TNF-a and IL-1b	Cytokine Quantification Real-Time PCR Flow cytometry	- BALB/c mice	- CT26 cells	[78]
Metastasis	Colon cancer	Not specified	Inflammasome pathway	IL-18 IL-1 Hepatic NK cells	Colon Liver Spleen	- Mice deficient in Caspase-1- were susceptible to CRC liver metastasis - Nlrp3 inflammasome is required to suppress CRC liver metastasis - IL-18 is critical for inflammasome mediated CRC growth in the liver suppression through the modulation of NK cells	Quantitative Real-Time PCR Flow cytometry Immunofluorescence staining	- C57BL6/J mice		[79]
	Colon cancer	*Fusobacterium nucleatum*	*Fusobacterium nucleatum*	CD8 T cells CD33 cells CD163 cells	Colon Liver	- The presence of F. nucleatum was associated with a lower CD8+ T cell density - The presence of F. nucleatum was associated with a higher myeloid-derived - suppressor cells densities (MDSC) - F. nucleatum promotes the development of colonic neoplasia through the recruitment of MDSCs into the tumor	Immunohistochemical staining DNA extraction Quantitative Real-Time PCR Immunohistochemistry	- Patients undergoing chemotherapy - ApcMin mouse	- Colorectal cancer liver metastases cells	[80]
	Colon cancer	*Firmicutes* *Proteobacteria*	Sodium butyrate	IL-10 IL-17 Hepatic NK cells	Colon Liver	- Sodium butyrate administration reduced Treg frequencies - Sodium butyrate significantly increased the rate of natural killer T cells in the liver - Sodium butyrate decreased IL-10 production while increasing the production of IL-17 in colorectal liver metastasis mice	Quantitative Real-Time PCR Hematoxylin and eosin stain Flow cytometry	- BALB/c mice		[81]
Apoptosis	Colon cancer	*Erysipelotrichaceae* *B.fragilis*	Follicular helper T (T_FH_) cells	caspase-3 caspase-7	Colon	- Ileal microbiota is critical for the activation of TFH cells - The density of TFH cells correlated with ileal caspase-3 activation during ileal apoptosis, suggesting a potential anti-tumor activity - Microbial structures such as bacterial RNA can trigger IL-1β-dependent differentiation of TFH cells.	Antibiotic treatment Flow cytometry Fecal microbiota transplantation ELISA 16S rRNA gene sequencing Immunohistochemistry staining	- C57BL/6J mice	- Luminal content from proximal colon - CT26 cells - 4T1 cells	[82]
	Colon cancer	*Bacteroides* *Firmicutes* *Prevotellaceae* *Lactobacillaceae*	Fucoidan	β-catenin C-Myc CyclinD1 IL-17 IL-23 Il-4 Il-10	Colon tissues	- Treatment with fucoidan increased cellular apoptosis and decreased tumor incidence and mean weight - Treatment with fucoidan decreased the expression of β-catenin C-Myc and CyclinD1 - The level of NK cells, interferon-γ, IL-4, IL-10, and CD4 T cells were increased in the fucoidan treated models, while the levels of interleukin (IL)-17 and IL-23 were decreased	Flow cytometry Western blotting Immunofluorescence assay 16S rRNA gene sequencing Gas chromatography	- Sprague–Dawley (SD) rats		[83]
	Colon cancer	Not specified	BCL-G (BCL2L14)	IFN-γ TNF-α	Colon	- BCL-G S/L level was upregulated during Th1 cytokine-induced apoptosis through the synergetic regulation of IFN-γ and TNF-α. - Both STAT1 and SWI/SNF-mediated chromatin remodeling played a role in the induction of BCL-G S/L level - Despite these results, BCL-G was unessential for death in intestinal epithelial cells	Crystal violet staining Microscopy Western blotting Chemokine analysis		- HT-29 cells - Colonic biopsy	[84]

### 4.1. Inflammation 

Inflammation is associated with multiple diseases such as diabetes, cardiovascular disease, and multiple stages of cancer [85]. During cancer, acute inflammation is critical in the recruitment and accumulation of neutrophils, the stimulation of antigen presentation, and the maturation of dendritic cells leading to an anti-tumor response. Additionally, and during acute inflammation, the level of C-reactive protein and serum amyloid A protein (SAA), acute phase proteins can increase, with the latter being influenced by segmented filamentous bacteria. On the other hand, chronic inflammation is linked to different stages of cancer development, including transformation, promotion, proliferation, invasion, metastasis, survival, angiogenesis, and treatment resistance, with an accumulation of macrophages, lymphocytes, and plasma cells at the site [86]. In addition, chronic inflammation is considered a risk factor for gastrointestinal cancer development in patients with inflammatory bowel disease, as reports have illustrated a similar inflammatory microenvironment between cancer and inflammatory bowel diseases. Additionally, in both diseases, inflammatory cells produce similar mediators such as IL-6 and IL-12, which suggests the role played by the immune system in both diseases [68]. Damaged tissues in the body caused by either a physical or an ischemic injury, exposure to toxins, or an infection can result in an inflammatory response activation that is necessary to repair the damaged tissues [87]. An inflammatory response can become chronic when the causative agent of the inflammation persists, resulting in cellular proliferation and mutation, thus creating a suitable environment for cancer development [88]. Additionally, and due to chronic inflammation, host leukocytes such as macrophages, dendritic cells, and lymphocytes can be present in tumor areas. They can lead to immunosuppression and cancer growth by producing reactive oxygen species (ROS) that damage the intestinal epithelial cells’ DNA [87]. 

**Figure 3:**
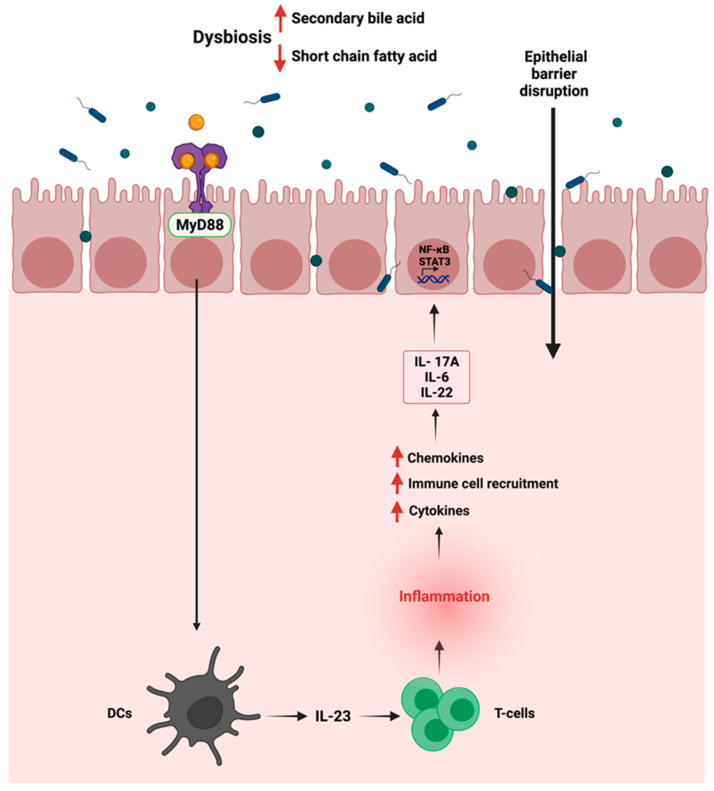
Schematic representation of the immune—gut interactions during GI cancer and how it influences the inflammatory responses. Due to gut dysbiosis, the low level of short chain fatty acids can lead to the activation of inflammatory pathway, the production of cytokines and chemokines and the activation of STAT3 and NF-kB signaling pathways. “Created with BioRender.com”.

The gut microbiota in the intestine is usually segregated from the immune cells by a single layer of intestinal epithelial cells joined by tight junctions [89]. Dysbiosis in the gut can alter the permeability of the intestinal barrier, causing a disruption where commensal bacteria and their products can invade the mucosa, thus resulting in low-grade systemic inflammation. Due to that, inflammatory pathways such as Wnt and Notch are activated, affecting the mucosal epithelial cells, thus influencing immune homeostasis and increasing susceptibility to CRC [90]. After activating the myeloid differentiation factor 88 (MyD88), the invading commensal bacteria and their products interact with TLRs on tumor-infiltrating myeloid cells, leading to the production of inflammatory cytokines such as IL-23 activating the production of IL-6, IL-22, and IL-17A [91]. The production of those cytokines can eventually promote the activation of STAT3 and the nuclear factor-kB (NF-kB) signaling pathway [92]. The promoted activation of NF-kB signaling pathway by TLR-4 overexpression can induce COX-2 expression, a CRC biomarker, and an inflammation-associated gene in inflammatory bowel disease [93]. Figure 3 summarizes the interaction between the gut microbiome and the immune cells in GI cancer and its activation of inflammation. Meanwhile, another preclinical study documented that the activation of the inflammatory response significantly correlated with the disturbance of the gut microbiota and changes in the fecal metabolites [94]. The authors found that these changes could be closely related to the occurrence of precancerous lesions of GC. The correlation analysis between inflammatory cytokines and gut microbiota/feces metabolites was evaluated in a N-methyl-N′-nitro-N-nitrosoguanidine multiple factors-induced rat model of GC. The results demonstrated a significant increase in pro-inflammatory serum cytokines such as IL-1β, IL-4, IL-6, IL-10, IFN-γ, TNF-α, and M-CSF.

On the other hand, there was a significant decrease in the level of chemokine (C-X-C motif) ligand 1 (CXCL1) in the model group vs. controls. In this regard, the gut microbiota and fecal metabolic phenotype composition in the model group revealed that *Lactobacillus* and *Bifidobacterium* significantly increased. At the same time, *Turicibacter*, *Romboutsia*, *Ruminococcaceae_UCG-014*, *Ruminococcaceae_UCG-005*, and *Ruminococcus_1* were significantly decreased compared to the control animals.

### 4.2. Cellular Proliferation 

Cellular proliferation is a fundamental process essential for the development and hemostasis of the organism [95]. It is tightly regulated to ensure a precise and complete genome duplication [96]. Multiple factors, from DNA damage to growth factors, influence the process of DNA replication, especially the entering to the S phase of the cycle [97]. Cancer cells embody multiple characteristics that play a role in their survival and abnormal proliferation [98] and due to epigenetic changes and/or mutations, cancer cells are resistant to cellular proliferation regulators such as growth factors and hormones. Such changes promote the growth and survival of cancerous cells through the stimulation of proliferation pathways and the inhibition of apoptotic pathways [99]. Emerging evidence supports the gut microbiome’s role in influencing cellular proliferation in cancer through contact with immune cells, as seen in the case of *Fusobacterium nucleatum*, the most studied colon cancer-associated microorganism, which is enriched during cancer [100,101]. 

*F. nucleatum* is a commensal opportunistic anaerobic Gram-negative bacillus found mainly in the oral cavity. It is implicated in multiple diseases outside the oral cavity [102]. *F. nucleatum* plays a role in colon cancer progression and treatment with antibiotics such as metronidazole which reduces their load and cellular proliferation [103]. Additionally, *F. nucleatum* promotes cellular proliferation in CRC by binding FadA to E-cadherin, which mediates the bacteria’s attachment and invasion. This leads to the activation of β-catenin signaling and the increased expression of Wnt genes, transcription factors, and inflammatory genes, thus impacting T-cells infiltration levels [104,105]. On the other hand, some bacterial strains, such as *Holdemanella biformis*, are reduced during gut tumorigenesis, which is critical in blocking tumor proliferation [106]. *H. biformis* impacts cellular proliferation by mediating SCFA such as butyrate, which inhibits histone deacetylase (HDAC) activities by enhancing H3 histone acetylation and reducing the NFATC3 pathway [107]. 

**Figure 4:**
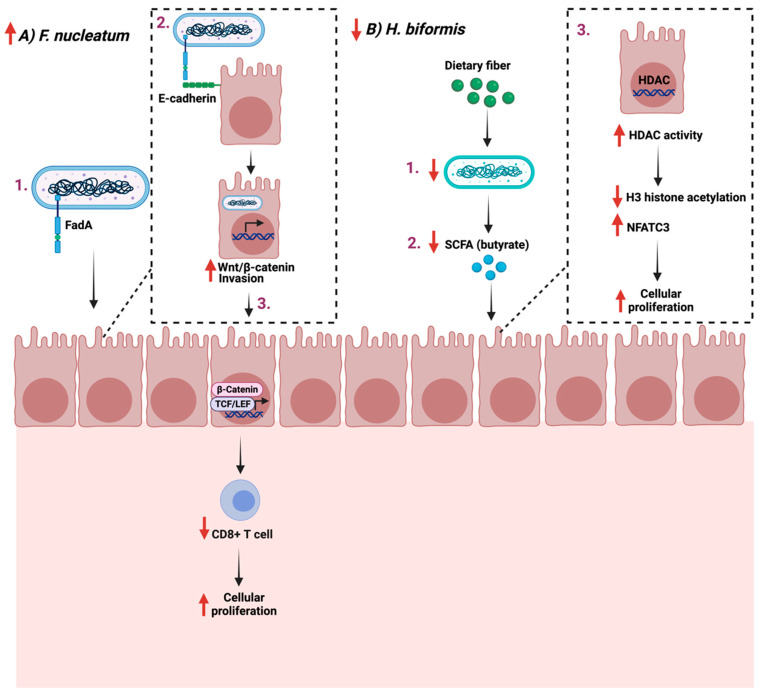
Schematic illustration of two pathways in which two bacteria *Fusobacterium nucleatum* and *Holdemanella biformis* facilitate cancer progression and cellular proliferation through FadA- E-cadherin interaction and short chain fatty acids (SCFA), respectively. (**A**) represent the proliferative example while (**B**) the anti-proliferative example. “Created with BioRender.com”.

Efforts are required to identify potential bacteria strains and their role in GI cancer development. Additionally, more research is necessary to assess the feasibility of maybe using specific strains as a treatment option for GI cancer. Figure 4 summarizes the role of the reported bacteria on GI cancer. 

### 4.3. Metastasis 

Metastasis is defined as the expansion of the primary tumor, leading to secondary tumors distant from the original tumor [108]. Metastasis occurs in a multi-step process that includes the separation from the primary tumor, the invasion through the surrounding tissues, and the entry and survival in the circulation [109]. Understanding the mechanism of metastasis is of great importance to managing and treating cancer. Therefore, assessing the impact of the gut microbiome, a potential therapeutic option, and immune system interaction can provide some insights. *F. nucleatum* is linked to CRC development and progression [110]. The polymerase chain reaction quantification of *F. nucleatum* DNA in 181 colorectal cancer liver metastases specimens reported that the presence and the quantity of the bacteria is inversely associated with a lower CD8+ T-cells density. This could suggest the potential involvement of *F. nucleatum* in cancer metastasis (Table 1) [80]. Mechanistically and in CRC, tissues are overexpressing sugar residues Gal-GalNAc, which is recognized by the *F. nucleatum* adhesion molecule, Fab2, and which is critical in mediating hemagglutinin and co-aggregation functions. Mechanistically, *F. nucleatum* could promote metastasis by activating the TLR-4 pathways, upregulating a cytochrome p450 known as CYP2J2. The metabolite of this cytochrome, 12,13-EpOME, then activates EMT, thus promoting CRC metastasis in vitro [111]. 

**Figure 5:**
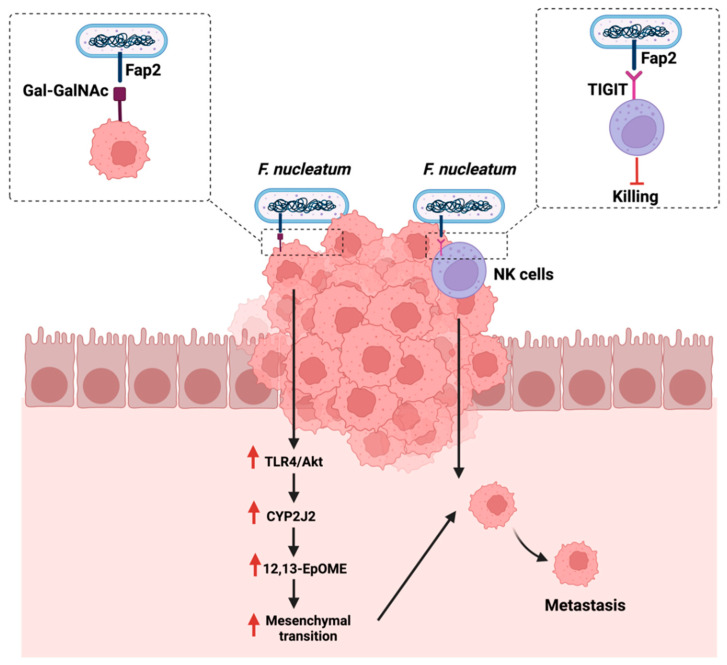
Summarizes the influence of *Fusobacterium nucleatum* on cancer metastasis either by targeting sugar residues Gal-GalNAc on cancerous cells or targeting receptors that are overexpressed on natural killer cells (NK). “Created with BioRender.com”.

Additionally, *F. nucleatum* can evade anti-cancer immune responses by mediating the recognition and binding of the same Fab2 adhesion molecule to a receptor known as TIGIT, overexpressed on natural killer cells and other lymphocytes. The mediated binding inhibits the functions of lymphocytes and natural killer cells, therefore, protecting *F. nucleatum* and promoting a pro-tumorigenic environment [112]. Figure 5 highlights the reported mechanisms in which *F. nucleatum* promotes GI cancer metastasis. 

### 4.4. Apoptosis 

Apoptosis is a basic cellular mechanism that is essential in the development and homeostasis of the organism [113]. Distinct morphological changes characterize it, controlled by intracellular and extracellular signals regulated by the cell environment [114]. Intrinsic and extrinsic pathways are the two major apoptotic pathways where they process the stress signal and execute the death signal in the cell [115]. Both exogenous and endogenous agents such as physical trauma, infectious agents, radiation, and chemotherapeutic drugs can trigger apoptosis [116]. In cancer, downregulation of apoptosis by pro-survival proteins is necessary to maintain the phenotypic properties. Such alteration is observed in the anti-apoptotic Bcl-2 family, which is overexpressed frequently in solid tumors [117]. On the other hand, a study analyzed the expression of human BCL-G, a member of the BCL-2 family in gastrointestinal conditions, and they reported that both variants were highly expressed in a healthy gut. At the same time, their m-RNA level was decreased in colorectal cancer and inflammatory bowel disease conditions [84]. Additionally, the study reported that the depletion of BCL-G affected the secretion of chemokines such as CCL5 thus illustrating a non-apoptotic function of the BCL-2 family. More studies are required to assess the role of the BCL-2 family in shaping the immune system, apoptosis, and maybe the regulation of chemokines (Table 1). 

The gut microbiome is a critical mediator of the host’s health by producing certain metabolites essential for immune system regulations [118]. Gut dysbiosis can reduce the beneficial bacteria responsible for producing SCFA, such as butyrate [119]. Butyrate plays a role in maintaining the intestinal barrier function and reducing inflammation in the colon, as they supply colonocytes with 70% of their required energy [120]. Additionally, the butyrate induces IL-18 expression in the colon, which is essential in suppressing colonic inflammation [121]. The administration of butyrate reduces cellular proliferation and pro-inflammatory cytokines production, such as IL-6, while promoting apoptosis [122]. Gut analysis of patients with colon cancer and ulcerative colitis showed a significant reduction in butyrate levels and the number of butyrate-producing bacteria in the colon [123]. During cancer, and when the gut is in dysbiosis, butyrate production is reduced, impacting the butyrate receptor’s activity, GPR109a, found in the colon. This reduces IL-18 and IL-22 production, reducing the mucosal tissue repair capabilities, thus impacting cellular apoptosis [124,125]. Another study described the significant role of moxibustion, a traditional Chinese medicine, in inducing apoptosis of rat GC cells in vivo by regulating intestinal flora [126]. The authors summarized that moxibustion delayed the GC metastasis possibly by lowering the abundance of *Ruminococcaceae* and *Prevotellaceae* bacteria (bacteria producing short-chain fatty acids in the gut) and enhancing the occurrence of probiotic *Akkermansia* in the rat intestine.

**Figure 6:**
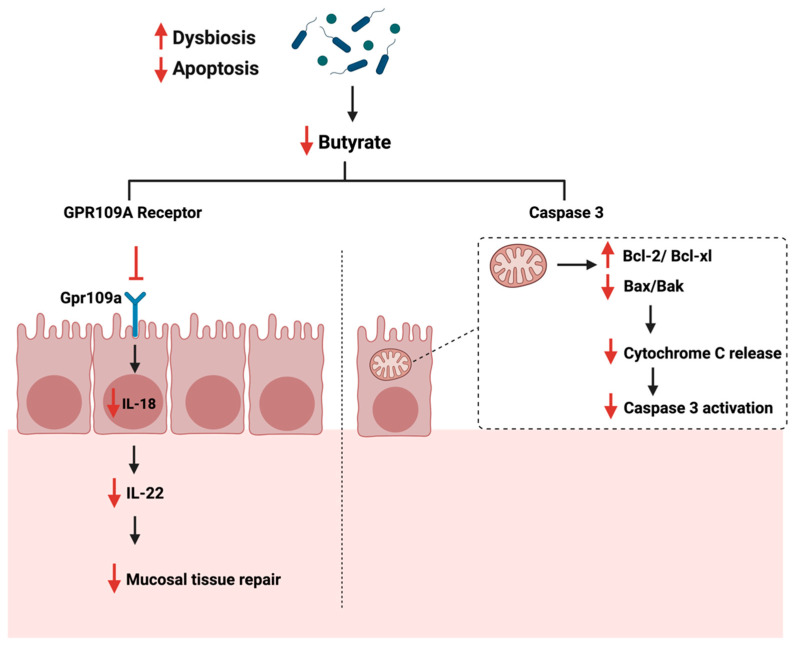
Illustrations of the role of short-chain fatty acids specifical butyrate on cellular apoptosis. The figure highlights the Gpr109a receptor and the pathways that lead to the reduction of mucosal tissue repair and Caspase 3 activation. “Created with BioRender.com”.

Additionally, butyrate induces apoptosis in CRC through the mitochondrial pathway and caspase 3 [127]. When the butyrate level is reduced, the expression of Bcl-2 anti-apoptotic family is enhanced, while the expression of Bax/Bak, cytochrome c is reduced [120]. Figure 6 summarizes the role of gut dysbiosis and butyrate production on cellular apoptosis during cancer. 

## 5. Discussion

### 5.1. Influence of Gut Microbiome on Immunotherapy

Current cancer treatments, including chemotherapy, surgery, endocrine therapy, and radiotherapy, are usually non-specific approaches. They frequently reach a refractory period, leading to treatment failure and disease recurrence [128,129]. Targeting the immune system and enhancing the patient’s immune system to attack the tumor can potentially be therapeutic [130]. Cancer immunotherapy is an alternative approach that utilizes specific components of a patient’s immune system to selectively target and eliminate tumor cells, thus mitigating the side effects of the currently used treatments [131]. Depending on the mechanism by which the therapy activates the immune response, immunotherapy can be passive, such as cell-based therapy and chimeric antigen receptor T cell therapy (CAR-T cell) or active, such as vaccination, immunostimulatory cytokines, and immune checkpoint inhibitors [132,133]. Immune checkpoint inhibitors are used as a treatment option to induce a T-cells mediated response against cancerous cells to selectively block the inhibitory checkpoint receptors manipulated by the tumor cell [134]. Types of inhibitory checkpoint receptors include programmed cell death protein 1 (PD-1), cytotoxic T lymphocyte-associated antigen 4 (CTLA-4), T cell immunoglobulin and mucin protein 3 (TIM-3), and programmed cell death 1 ligand 1 (PD-L1) [135]. To treat CRC, immunomodulatory therapy such as CTLA4, PD-1, and PD-L1 is currently used to target selective checkpoint molecules and inhibit T-cell activation [136]. Despite this, 19 patients with unselected CRC did not demonstrate positive clinical responses when using Nivolumab, a monoclonal antibody that binds to PD-1 receptor [137]. 

**Figure 7:**
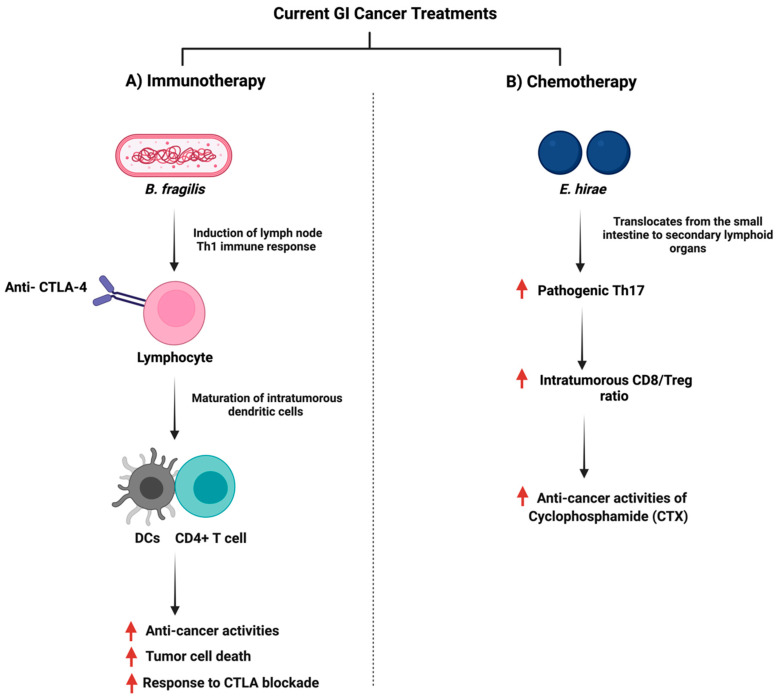
Illustrations of the gut bacteria and their role in modulating the efficacy of the currently used anti-cancer drugs. The figure summarizes the influence of the reported bacteria on immunotherapy and chemotherapy treatments. “Created with BioRender.com”.

The gut microbiome plays a role in stimulating and influencing immunotherapy against cancer [138]. The intestinal microbiota is an essential factor in providing an optimal CpG-oligonucleotide immunotherapy response which activates innate immune cells [139]. Moreover, the microbiome influences immunotherapy as a community, but specific microbes such as *Bacteroides fragilis* can enhance PD-1/PD-L1 and CTLA-4 immunotherapy as they activate Th1 cells [140]. Figure 7 summarizes the interaction of *B. fragilis* with immunotherapy. Additionally, in 74 advanced gastrointestinal cancer patients, the ratio of *Prevotella/Bacteroides* was elevated with an enhanced anti-PD-1/PD-L1 treatment response [141]. The analysis of DNA sequencing of stool samples collected before the administration of checkpoint inhibitors illustrated a distinct bacterial taxa composition [142], and that microbial species capable of producing SCFA were reported to have better anti-PD-1/PD-L1 positive responses [141]. A mice model study showed that *Prevotella* CAG:485 and *Akkermansia* might influence the efficacy of PD-1 immunotherapy through the modulation of glycerophospholipid metabolism, which can affect the expression of cytokines such as IL-2 and IFN-γ [143]. More clinical and experimental trials are necessary to investigate how the gut microbiome impacts immunotherapy. 

### 5.2. Chemotherapy Treatment and Immune–Gut Interactions

Chemotherapy is used as a treatment option for cancer, with platinum and fluorouracil being the commonly used drugs [144,145]. Regularly, cancer patients receiving chemotherapy have signs of depression, fatigue, anxiety, and cognitive impairment [146]. Chemotherapy treatment is often accompanied by multiple complications caused by the cytotoxic effect, linked to a bidirectional interaction between the drug and the gut microbiome [147]. Preclinical model studies demonstrated chemotherapy-induced changes in the gut microbiome with a decrease in the total number and diversity of the gut microbiome [148]. Additionally, and depending on the drug used, the overall impact on the gut profile reported a reduction in *Lactobacillus* and *Bifidobacterium*, and an increase in *Escherichia coli (E. coli)* and *Staphylococcus*. The reported gut microbiome composition disruption was associated with activating inflammatory pathways, thus enhancing the vulnerability to pathogenic infections [149].

On the other hand, the efficacy of chemotherapy can be affected by the gut microbiome. Such a mechanism includes when specific oral or injected drugs, such as CPT-11 (Irinotecan) depend on the gut microbiome to be converted to the active form and the treatment can exert anti-cancer properties [150]. Moreover, the gut microbiome can facilitate the anti-cancer effects of chemotherapy through the induction of enzymatic expression responsible for ROS production, which can induce cellular apoptosis [151]. Additionally, the gut microbiome can impact the ROS pathway through a toll-like receptor agonist, which can downstream the expression of MyD88 and induces inflammatory cytokines such as IL-6 [152,153]. Chemotherapy treatment and the gut microbiome can influence the immune system and changes in the gut microbiome due to chemotherapy can impact innate immunity by reducing the production of inflammatory cytokines and antigen-presenting cells [154]. For example, both *Enterococcus hirae* and *Lactobacillus johnsonii* were essential for the anti-cancer activities of Cyclophosphamide (CTX) where they promoted splenic Th1 memory and a Th17 response [155]. Figure 7 summarizes the interaction of *E. hirae* with CTX treatment. 

Although multiple reports illustrate the role of the gut microbiome in chemotherapy, some studies highlight microbiota-induced chemoresistance. The gut of patients with CRC is enriched with *F. nucleatum,* which was discussed in the above sections along with how it can promote metastasis [156]. This phylum can induce chemoresistance in which the inflammatory pathway is stimulated by the mediated binding of FadA and E-cadherin, which can then increase tumor growth [157]. Additionally, the gut microbiome can inactivate the used chemotherapy drug, inducing chemoresistance as seen with *Gammaproteobacteria*, which can convert the gemcitabine drug to its inactive metabolite, thus contributing to drug resistance [147]. All data indicate that efforts are required to investigate the bidirectional interaction between the gut microbiota and chemotherapy and the possibility of using this interaction to improve the treatment outcome further and reduce chemoresistance development. 

### 5.3. Challenges with Studying the Field

The area of the gut microbiome and immune interaction research is growing as scientists understand more about microbial communities, their behaviors, core microbial species, their produced metabolites, and their influence on the host immune system in health and disease as in the case of GI cancer. Despite this, the field faces multiple challenges, including protocol standardization, experimental models, and interpretation tools. Additionally, the gut is influenced by several factors such as diet, geographical location, genetic diversity, and medications, thus requiring a systematic and extensive data analysis. Moreover, investigating the mechanistic pathways in which the gut microbiome influences the immune response during cancer is critical as those interactions might provide potential therapeutic targets. Collective efforts from microbiologists, ecologists, bioinformaticians, immunologists, and geneticists are fundamental to improving the field further. 

### 5.4. Future of GI Cancer Treatment?

As discussed in the previous sections, the gut microbiome can interfere directly or indirectly with current treatments such as chemotherapy and immunotherapy, which might impact a treatment’s outcome. Manipulating the gut microbiome composition using fecal microbiota transplantation or phytochemicals might improve therapeutic outcomes [158]. Fecal microbiota transplantation (FMT) is known as the transplantation of microbes from the gut of a healthy donor to a recipient either through the upper or lower gastrointestinal tract [159]. It was first documented in clinical use in 1958 to treat *Clostridium difficile* infection as it helped treat 80% of the affected patients [160]. The advantages of using FMT include its safety and its ability to restore intestinal microbial diversity [161]. Limited studies are available in the literature that investigates the role and the application of FMT in the context of GI cancer treatment. We found a study that reported the effectiveness of FMT in mice receiving intestinal microbiota from wild mice, as the results showed better resistance to CRC [162]. Additionally, and on a different approach, the usage of phytochemicals for GI cancer treatment has recently gained attention. The bioactive plant-derived compounds generally have lower oral bioavailability due to poor aqueous solubility, and therefore, the gut microbiome is essential for the metabolism and absorption of bioactive compounds [163]. Several data support the role of 13 bioactive secondary compounds on GI cancer [164]. For example, lutein, an abundant fat-soluble bioactive compound found primarily in green leaved vegetables, was reported to significantly reduce aberrant crypt foci (ACF) in the colon of mice, thus reducing cellular proliferation [165]. Despite those reports that support potential treatments, research is much needed to investigate the potential synergetic effects between the currently used treatments and FMT or phytochemicals. Additionally, attention should be given to the required concentration and the appropriate delivery mode of FMT and phytochemicals to avoid toxicity and possible side effects. Moreover, looking at the role of gut enzymes in the metabolism and the utilization of those natural bioactive compounds, research is needed to investigate the underlying mechanisms played by those enzymes that might affect the treatment outcome, as we have shown in our recently published paper [166]. 

## 6. Conclusions

The gut microbiome plays an essential role in mediating the immune response, impacting its activities, development, and function. Generally, and during cancer, signature microbes in the gut influence the anti-tumor activities by producing specific metabolites or inducing T-cell responses. On the other hand, some reported bacterial species enhance cellular proliferation and metastasis during cancer and understanding those interactions in the context of cancer may provide potential therapeutic targets. Despite the advances in the field, more research is needed to understand the underlying mechanisms, investigate the impact on current treatments, and identify specific microbes and immune cells that might lead to this interaction. Additionally, clinical trials are essential to assess the influence of immune–gut interaction on immunotherapy treatment in clinical settings. 

## Abbreviations

GI	gastrointestinal
CRC	colorectal cancer
GC	gastric cancer
EC	esophageal cancer
HCC	hepatocellular carcinoma
NF-κB	nuclear factor kappa
EMT	epithelial-mesenchymal transition
FISH	Fluorescence in situ hybridization
PD-1	Programmed death ligand 1
PD-1	Programmed cell death protein 1
SFB	Segmented filamentous bacteria
SAA	Serum amyloid A
DC	Dendritic cell
AMP	Antimicrobial peptide
TLR	Toll-like receptor
SCFA	Short chain fatty acid
GPCRS	G-protein coupled receptors
HDAC	Histone deacetylase
ROS	Reactive oxygen species
*F. nucleatum*	*Fusobacterium nucleatum*
CTLA-4	cytotoxic T lymphocyte-associated antigen 4
TIM-3	T cell immunoglobulin and mucin protein 3
FMT	Fecal microbiota transplant
ACF	Aberrant crypt foci
